# Advanced hybrid machine learning models with explainable AI for predicting residual friction angle in clay soils

**DOI:** 10.1038/s41598-025-05962-6

**Published:** 2025-07-16

**Authors:** Mawuko Luke Yaw Ankah, Shalom Adjei-Yeboah, Yao Yevenyo Ziggah, Edmund Nana Asare

**Affiliations:** 1 Geological Engineering Department, University of Mines and Technology, P. O. Box 237, Tarkwa, Ghana; 2 Geomatic Engineering Department, University of Mines and Technology, P. O. Box 237, Tarkwa, Ghana

**Keywords:** Residual friction angle, Hybrid Machine learning, ExPlanable AI, GrowNet, Natural hazards, Engineering, Civil engineering

## Abstract

Accurately estimating the residual strength friction angle of clay soils is vital for the design and stability evaluation of geotechnical structures such as slopes, retaining walls, and foundations, especially in regions susceptible to landslides and ground instability. Traditional methods for determining the residual strength friction angle are often labor-intensive, time-consuming, and costly. This study explores three advanced hybrid machine learning models: Gradient Boosting Neural Network (GrowNet), Reinforcement Learning Gradient Boosting Machine (RL-GBM), and a Stacking Ensemble to predict the residual friction angle of clay soils, addressing a critical gap in current predictive methodologies. The research utilized a carefully harmonized dataset of 400 samples from global studies, considering various soil parameters (liquid limit, plasticity index, change in plasticity index, and clay fraction) as inputs for the prediction models. Various evaluation metrics, including coefficient of determination (R^2^), Root Mean Squared Error (RMSE), and Mean Absolute Error (MAE), were used to assess model performance. The results show that GrowNet achieved the largest R^2^ of 0.94 and the lowest RMSE and MAE values of 1.87 and 1.17 on the testing dataset, representing a substantial improvement over traditional empirical correlations and previous machine learning approaches. To address model transparency, Explainable Artificial Intelligence (XAI) techniques, including SHAP (Shapley Additive exPlanations) and LIME (Local Interpretable Model-agnostic Explanations), were applied to the GrowNet model. These techniques consistently identified Clay Fraction as the most influential variable, followed by Plasticity Index. Integrating high-performing machine learning models with interpretability tools significantly improves the accuracy and reliability of residual friction angle predictions, offering practical value for geotechnical engineering applications.

## Introduction

Residual shear strength (RSS) refers to the minimum shear strength mobilized at large displacements when soils are subjected to prolonged shear loading at a slow rate^1–7^. In recent decades, significant attention has been devoted to the RSS of clay soils due to its critical role in predicting landslide behaviour^8–10^. RSS is defined by the shear strength mobilized along a preexisting shear surface^11–13^. Such shear surfaces may form due to past landslide events, tectonic activity, groundwater fluctations, earthquake-induced forces, or dynamic impacts such as blasting^14–16^. The residual strength of clay soils is influenced by particle size distribution and the characteristics of both the solid matrix and the pore fluid^17,18^. At the residual state, significant shear strains disrupt interparticle bonds and obliterate the original fabric of the soil, resulting in a reoriented structure with reduced strength^19^.

Consequently, clay soils at the residual state are commonly assumed to behave as cohesionless materials, where apparent cohesion becomes negligible or effectively zero^11,20,21^. This marks a fundamental shift in shear strength behaviour. While intact clay soils derive their strength from both cohesion (c) and the friction angle (φ), after large displacements, cohesion is lost, and shear strength depends mainly on the residual friction angle (φ_r_). As such, residual shear strength becomes highly sensitive to remolding effects. Given this transformation in mechanical behaviour, the present study focuses on predicting the residual friction angle as it serves as the dominant contributor to the residual shear strength of clay soils.

Laboratory tests for determining residual shear strength are often expensive and time-consuming. In this regard, practical alternatives such as empirical correlations based on easily accessible index properties, including the liquid limit (LL) and clay-sized fraction (CF), have been widely adopted^11,22–24^. The cost of a standard ring shear test to determine residual shear strength parameters varies depending on geographic location, sample preparation requirements, and the testing facility’s equipment. As specialized equipment is often required, it may not be readily available in many laboratories. Typically, commercial laboratories quote prices ranging from $800 to $1200 per sample. In contrast, the soil index properties used in predictive models (such as liquid limit, plasticity index, and clay-sized fraction) can be obtained through routine soil classification tests that cost significantly less (approximately $150-$200 per sample) and are widely available. This substantial cost differential, coupled with the significant time savings (ring shear tests can take weeks, while classification tests typically take days), provides strong economic justification for developing accurate predictive models for residual shear strength parameters.

Several studies have explored the relationship between residual shear strength (RSS) and Atterberg limits, as well as the influence of clay content^25–30^. In addition, a strong correlation has been reported between RSS and the plasticity index (PI)^31,32^. Skempton^33^ was among the earliest to attempt to establish a correlation between RSS and the clay content (clay fraction), highlighting the significance of particle composition in governing shear strength behaviour. Furthermore, many studies have proposed empirical relationships between the residual secant frictional angle and the liquid limit (LL), underscoring its role as a critical predictor in clay soils^34–36^.

In recent years, various machine learning approaches have been employed to predict the residual secant frictional angle (*ϕ*_r_) of soils, as summarized in Table 1^37–40^. Tran et al.^37^ explored the use of an artificial neural network (ANN) architecture for predicting the *ϕ*_r_ of clay-dominated soil based on soil index properties. Their ANN model demonstrated a higher coefficient of determination compared to alternative methods. Similarly, Khan et al.^21^ utilised a Functional Network (FN) model to estimate the residual shear strength of clay soils using a dataset compiled from the literature. The performance of the FN model was compared to other models, including Support Vector Machine (SVM) and ANN, with the FN demonstrating superior accuracy. Also, Das et al.^40^ utilised machine learning techniques to forecast the residual shear strength of clay soils, employing selected soil index properties as inputs for various ANN models and an SVM model. Their study further proposed a mathematical prediction equation, with the SVM model outperforming the ANN models in terms of prediction accuracy. This superior performance was attributed to the SVM model’s capability to minimize generalization error.

**Table 1:** Summary of ML Models for Predicting the Residual Shear Strength (RSS) of Clay Soils.

References	ML model	Data size	Input Parameters	Accuracy of Model
Das et al.^40^	ANN, SVM	162	LL, PI, CF, ∆PI	73%, 80%
Gholami and Bodaghi^19^	ONN, OFL, CM	162	LL, PI, CF, ∆PI	93%, 94%, 95%
Khan et al.^21^	FN, SVM, ANN	162	LL, PI, CF, ∆PI	91.1%, 88%, 89.8%
Riahi-Madvar et al.^41^	POMGGP	262	LL, PI, CF, ∆PI	72%
Tran et al.^37^	ANN	82	LL, PI, A-Line value, CF, Massive Minerals	96%

Despite notable advances, several significant research gaps remain in the prediction of the residual shear strength of clay soils. First, although various machine learning models have been employed, hybrid approaches that leverage the complementary strengths of different algorithms have been relatively underexplored. Traditional models often struggle to capture the complex non-linear relationships between soil index properties and residual strength parameters. In addition, many previous studies have been constrained by limited datasets (typically fewer than 300 samples), which limits the generalizability and robustness of the resulting models. Moreover, there is a notable lack of interpretability in many existing machine learning models, making it difficult for geotechnical engineers to understand and trust the predictions.

To address these gaps, this study advances the prediction of residual shear strength of clay soils by implementing two advanced hybrid machine learning approaches alongside a stacking generalization framework to enhance model efficiency and predictive accuracy. Specifically, this study employs a Reinforcement Learning Gradient Boosting Machine (RL-GBM), a Gradient Boosting Neural Network (GrowNet), and a Stacking ensemble method to estimate the residual secant friction angle (ϕ_r_) of clay soils.

Given that machine learning models must provide transparent reasoning to be confidently adopted in engineering decision-making ^42^, this study also conducts model-agnostic interpretability analysis to assess the sensitivity of each input parameter on the predicted residual friction angle (ϕ_r_).

### Residual shear strength determination via ring shear test

Residual shear strength measurements derived from ring shear tests are considered more accurate and representative than those obtained from traditional direct shear or triaxial methods^43^.

These laboratory tests are commonly conducted in accordance with standard procedures such as those of the American Society for Testing and Materials (ASTM) with a fixed designation of D6467. Based on this procedure, a reconstituted, overconsolidated, and presheared specimen is sheared at a controlled displacement rate until a constant drained shear resistance is achieved along a single shear surface defined by the apparatus. During testing, the specimen rotates continuously in one direction, and displacement is calculated by multiplying the average specimen radius by the degrees of rotation, factoring in a conversion constant of 0.0174. Although intact or naturally sheared specimens can be tested, challenges in sampling, identifying the shear direction, and aligning non-horizontal surfaces make their use less practical. Therefore, the method primarily employs reconstituted specimens, enabling continuous shear displacement to reliably determine residual strength.

In determining residual shear strength via ring shear apparatus, the applied torque (T) on the annular sample is measured, and shear stress (τ_r_) is calculated using the equation τ_r_ = T/2πR^2^Δr, where R is the mean radius of the annular specimen and Δr is the radial thickness. The normal stress (σ_n_) is calculated by dividing the normal force by the sample area. Multiple tests at different normal stresses are conducted to establish the residual failure envelope, which is described by the Mohr–Coulomb criterion: τ_r_ = c_r_ + σ_n_·tan(φ_r_), where c_r_ represents residual cohesion (which typically approaches zero), and φ_r_ is the residual friction angle. The value of tan(φ_r_) represents the coefficient of residual friction, and φ_r_ itself is the residual internal friction angle of the soil after large displacements have occurred and particles (especially clay minerals) have reoriented along the shear plane. Since the residual cohesion (c_r_) typically approaches zero after large displacements, the friction angle becomes the defining parameter.

## Methodology

**Fig. 1:**
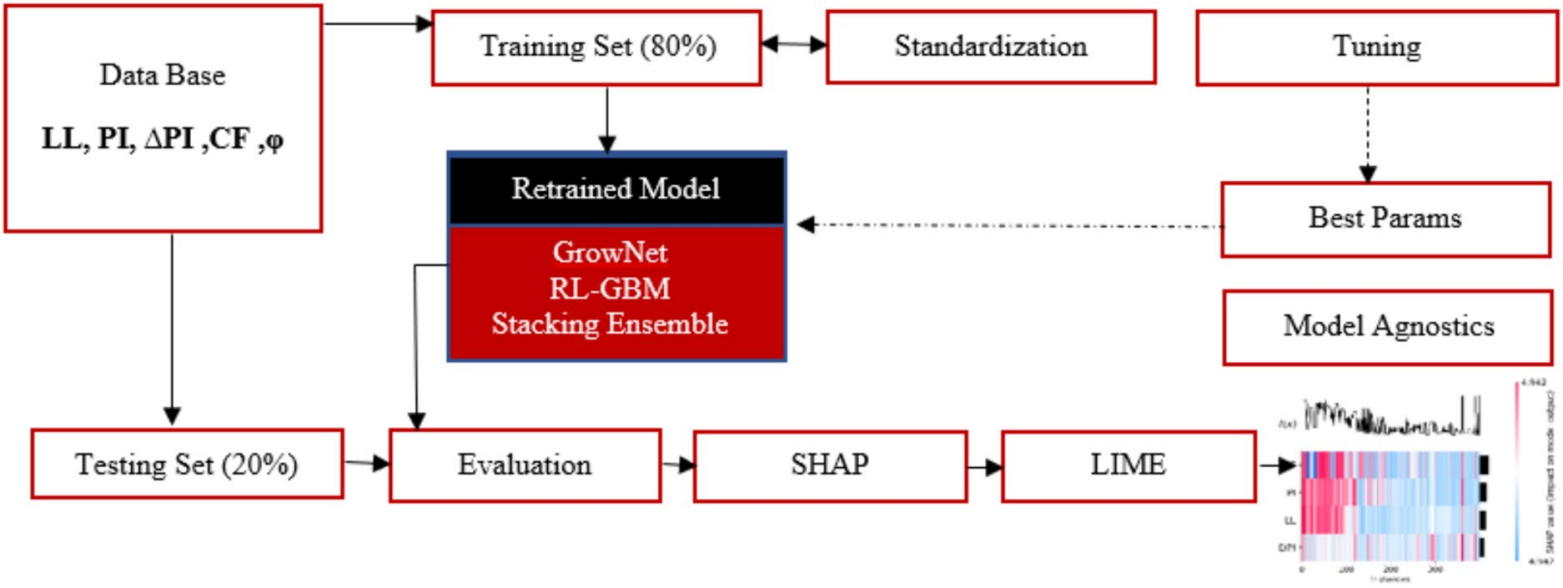
Flowchart of the Research Framework for Predicting Residual Shear Strength (RSS) of Clay Soils.

As mentioned earlier, the study employed two advanced hybrid ensemble learning techniques (RL-GBM and Grownet) and a stacking ensemble framework to predict the RSS of clay soils. A summary of the research workflow is presented in Fig. 1.

### Data acquisition and preprocessing

A curated dataset consisting of 400 data points compiled from previous studies was utilised to train and test the models^23,40,41,44,45^. The soil samples analyzed in the abovementioned studies were sourced from a range of geological and geographical regions, including areas affected by landslides, debris flows, slope failures, and volcanic eruptions. The residual friction angle (RFA) measurements from these laboratory investigations were evaluated using a laboratory ring shear device, with mean values considered for analysis. To ensure data reliability and consistency, a rigorous data harmonization process was implemented. This included: standardization of measurement units across all studies, verification of data points against source publications, removal of outliers identified through statistical analysis (z-score), and normalization of data to account for variations in testing conditions.

The dataset was partitioned using an 80/20 train-test split ratio. This ratio was selected based on previous research recommendations^46,47^. For a dataset of 400 samples with 4 input features, an 80% training allocation (320 samples) provides sufficient data to train robust models while minimizing the risk of overfitting. The remaining 20% (80 samples) provides an adequate test set to evaluate model generalization without sacrificing training data quality. This split represents an optimal balance for geotechnical data modeling based on similar successful applications in the literature.

#### Input feature selection

The selection of input features was guided by established soil mechanics theory and supported by empirical findings from prior studies. Four key soil parameters were considered for model development. The Liquid Limit (LL), Plasticity Index (PI), Clay Fraction (CF), and differential plasticity index (∆PI). These parameters are the primary variables commonly used by researchers in predicting the residual shear strength of clay soils^19,37,41^.

The Liquid Limit (LL), which represents the water content at which soil transitions from a plastic to a liquid state, has been consistently associated with residual strength properties^34,35^. The Plasticity Index (PI), defined as the range of water content over which soil exhibits plastic behavior, reflects the mineralogical characteristics of clay and has demonstrated strong correlations with the residual friction angle^31,32^. The Clay Fraction (CF), representing the percentage of soil particles smaller than 0.002 mm, is widely recognized as a dominant factor influencing residual shear strength^29,33^. Finally, the Change in Plasticity Index (ΔPI), calculated as PI − 0.73(LL − 20), quantifies the deviation from the A-line in the Casagrande plasticity chart and captures additional information about clay mineralogy not fully described by LL and PI alone.

#### Data statistics

**Fig. 2:**
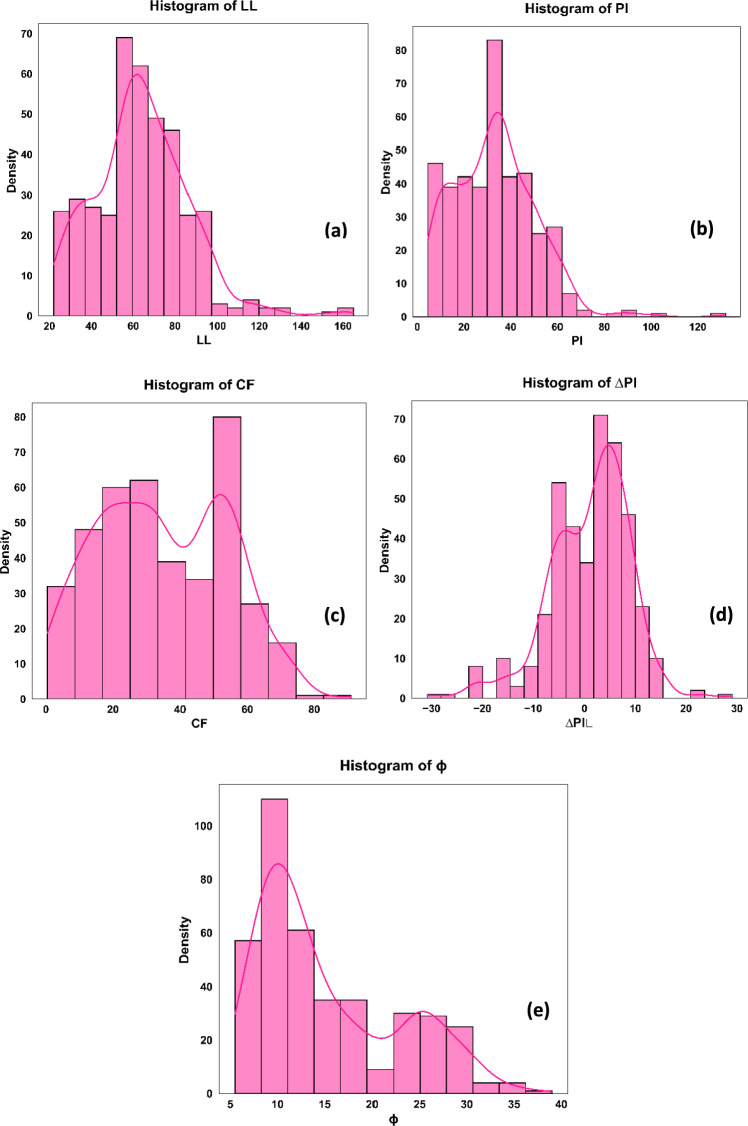
Frequency Histograms of the Various Parameters: (**a**) ΔPI; (**b**) LL; (**c**) PI; (**d**) CF; and (**e**) ϕ.

**Fig. 3:**
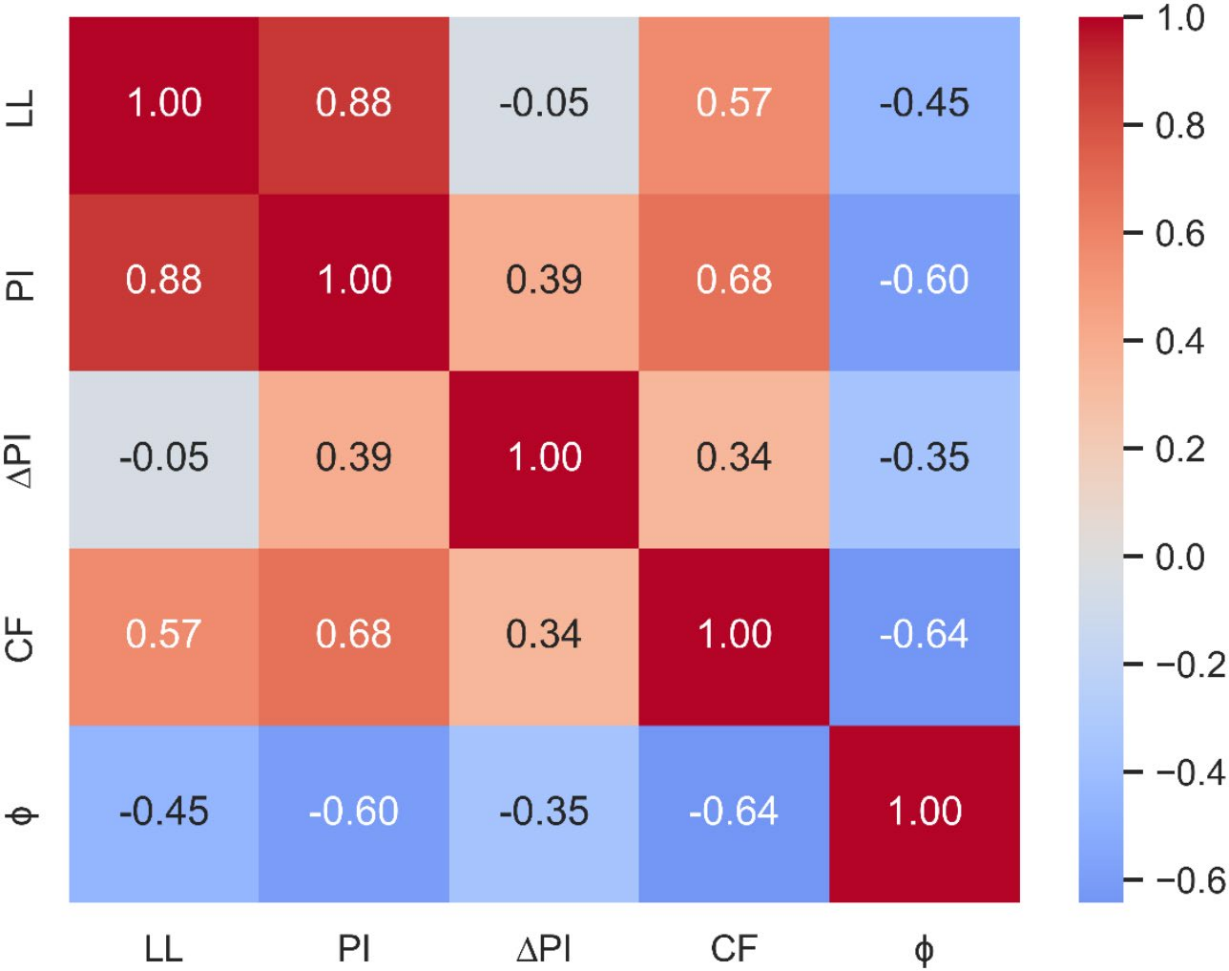
Spearman’s Correlation Coefficient of Variables.

All 400 data points used in this study include the selected soil index properties: Liquid Limit (LL), Plasticity Index (PI), Change in Plasticity Index (∆PI), and Clay Fraction (CF) as input variables (covariates), with the residual friction angle (ϕ_r_) serving as the dependent variable. Descriptive statistics of the dataset are presented in Table 2, while the distribution of each feature is illustrated through frequency histograms in Fig. 2. The Spearman correlation matrix (Fig. 3) provides insights into the monotonic relationships between each covariate and ϕ_r_. To enhance model training reliability, all input features were standardized using Eq. (1).

1 $${X}_{std}=\frac{X-\mu }{\sigma }$$

where X is the feature value, $\mu$ is the mean of the feature variable, and $\sigma$ represents the standard deviation of the feature variable.

**Table 2:** Descriptive Statistics of Input Variables for Residual Friction Angle Prediction.

Description	(LL)%	(PI)%	(∆PI)%	(CF)%	(*ϕ*_r_) °
Minimum	22.00	4.50	-30.85	0.40	5.50
Mean	63.71	32.93	1.16	34.9	15.29
Standard Deviation	22.44	17.59	7.81	19.07	7.38
Max	165.00	132.00	29.07	91.00	39.00

The Spearman correlation matrix (Fig. 3) illustrates the strength and direction of monotonic relationships between pairs of variables. A larger absolute value of the Spearman correlation coefficient (r_s_) indicates a stronger association, while smaller values suggest weaker relationships. As shown in Fig. 3, the residual friction angle (ϕ_r_) exhibits a strong negative correlation with Clay Fraction (CF) (r_s_ = -0.64) and a strong positive correlation with Plasticity Index (PI) (r_s_ = 0.60). It also shows a moderate negative correlation with Liquid Limit (LL) (r_s_ = -0.45) and a weak negative correlation with Change in Plasticity Index (∆PI) (r_s_ = -0.35). These results indicate that the clay fraction has the most pronounced inverse effect on ϕ_r_, with the other features also contributing to a reduction in the friction angle as their values increase.

#### Multicollinearity considerations

One of the key advantages of advanced machine learning models is their inherent ability to manage correlated input features more effectively than traditional approaches. In this study, we addressed potential multicollinearity among the input variables by employing a combination of regularized hybrid models (GrowNet and RL-GBM) as well as a stacking ensemble framework. The hybrid models inherently mitigate multicollinearity through their architectural designs: GrowNet integrates neural network layers with built-in regularization, while RL-GBM uses dynamic learning rate adjustments to control model complexity and reduce the influence of redundant features.

To further enhance predictive robustness, we adopted a stacking ensemble technique which combines outputs of multiple base learners to generate a meta-model. This approach leverages the strengths of individual models while reducing overfitting and the impact of multicollinearity. By integrating diverser learning algorithms, each with its own inductive bias and regularization scheme, the stacking ensemble provides a more stable and generalized prediction framework.

### Gradient boosting neural networks (GrowNet)

GrowNet introduces a neural network architecture inspired by gradient boosting techniques. It dynamically adapts its configuration based on the characteristics of the input data^48^. To overcome the limitations of greedy function approximation in conventional gradient boosting decision trees, GrowNet incorporates a fully corrective step^49^. This approach combines the predictive power of gradient boosting with the flexibility and learning capacity of neural networks^50^.

The GrowNet approach employed in this study involves a sequential hybrid learning framework that integrates gradient boosting and neural networks for enhanced prediction of the residual friction angle. Initially, the standardized dataset was partitioned into training and testing subsets to ensure robust model evaluation. An XGBoost regressor was first trained as the base model, incorporating hyperparameter tuning and regularization to optimize its performance. Following this, the prediction residuals, representing the errors from the XGBoost model, were computed for both the training and testing sets.

These residuals were subsequently used to train a neural network, which was designed to learn and correct the patterns not captured by the initial XGBoost model. The neural network was optimized using the Adam optimizer with a learning rate of 0.005 and mean squared error as the loss function. To mitigate overfitting, an early stopping strategy was employed, terminating training if the validation loss did not improve for 15 consecutive epochs. The model with the best validation performance was preserved for final prediction. Ultimately, the final output was obtained by aggregating the predictions from both the XGBoost and neural network components, thereby enhancing the overall accuracy and generalization capability of the GrowNet architecture.

Consider a residual friction angle dataset consisting of *n* samples within a *d*-dimensional feature space, denoted as $\mathcal{D}={\left\{\left({\mathbf{x}}_{i},{y}_{i}\right)\right\}}_{i=1}^{n},{\mathbf{x}}_{i}\in {\mathbb{R}}^{d},{y}_{i}\in {\mathbb{R}}\}$, where *x*_i_ ∈ ℝ^d^ represents the input feature vector and *y*_i_ ∈ ℝ denotes the corresponding residual friction angle. GrowNet models the target variable using an ensemble of *K* additive functions, such that the predicted output is given by:

$${\widehat{y}}_{i}=\mathcal{E}\left({\mathbf{x}}_{i}\right)=\sum_{k=0}^{K} {\alpha }_{k}{f}_{k}\left({\mathbf{x}}_{i}\right),{f}_{k}\in \mathcal{F}$$

$\mathcal{F}$ represents the function space of shallow neural networks and ${\alpha }_{k}$ is the boosting step size. Each weak learner ${f}_{k}$ is trained to correct the residuals from the preceding ensemble model. The objective is to learn a set of functions that minimize the total loss: $\mathcal{L}(\mathcal{E})=\sum_{i=1}^{n} {\ell}\left({y}_{i},{\widehat{y}}_{i}\right)$. At stage *t*, the current ensemble prediction is: ${\widehat{y}}_{i}^{(t-1)}=\sum_{k=0}^{t-1} {\alpha }_{k}{f}_{k}\left({\mathbf{x}}_{i}\right)$. The next weak learner ${f}_{k}$ (x) is then trained to minimize the loss function at stage *t* defined as:

2 $${\mathcal{L}}^{(t)}=\sum_{i=1}^{n} {\ell}\left({y}_{i},{\widehat{y}}_{i}^{(t-1)}+{\alpha }_{t}{f}_{t}\left({\mathbf{x}}_{i}\right)\right)$$

To prevent overfitting and penalize model complexity, ${{\ell}}_{1},{{\ell}}_{2}$ regularization terms are added^51^. Additionally, a Taylor expansion of the loss function ${\ell}$ is employed to reduce computational complexity and facilitate efficient optimization.

### Reinforcement learning gradient boosting

Reinforcement Learning Gradient Boosting (RLGB) is a novel framework that extends the interpretability, efficiency, and predictive performance of Gradient Boosted Trees within the Reinforcement Learning (RL) domain^52,53^. Unlike conventional techniques such as hyperparameter optimization, ensemble blending, or neural architecture search, RLGB integrates reinforcement learning directly into the gradient boosting process. The RLGB framework incorporates a Q-learning agent to dynamically select the learning rate at each boosting iteration.

 Let ${a}_{t}$ denote the standard learning rate determined by the boosting algorithm. In RLGB a reinforcement learning agent adaptively selects a scaling factor from the set $\mathcal{A}=\left\{\text{0.5,0.7,1.0,1.3,1}.5\right\}$ to modify ${a}_{t}$ at each iteration. The algorithm begins with an initial ensemble prediction ${\widehat{F}}_{0}$, typically the mean of the training targets. At each iteration *t*, a decision tree is trained to model the residuals ${g}_{t}=y-{\dot{F}}_{t-1}$, where *y* denotes the observed residual friction angles (*ϕ*_r_). The reinforcement learning (RL) agent observes the environment through a discrete state representation $s_{t} = min\left( {\left\lfloor {err_{t} \times 10} \right\rfloor ,9} \right)$, where $er{r}_{t}$ is the mean absolute residual at iteration *t*. Based on ${s}_{t}$, the agent selects a learning rate modifier ${a}_{t}\in \mathcal{A}$ using an $\in$-greedy policy: with probability $\in$, the agent explores by selecting a random action from $\mathcal{A}$; otherwise, it exploits the current knowledge encoded in a Q-table $Q(s,a)$. After updating the model, the agent receives a reward ${r}_{t}$, defined as the reduction in mean absolute percentage error (MAPE) on the training set. It then updates its Q-values using the standard Q-learning update rule:

$$Q\left({s}_{t},{a}_{t}\right)\leftarrow Q\left({s}_{t},{a}_{t}\right)+{\alpha }_{q}\left[{r}_{t}+{\gamma }_{q}\underset{a}{max} Q\left({s}_{t+1},a\right)-Q\left({s}_{t},{a}_{t}\right)\right]$$

where ${\alpha }_{q}$ is the Q-learning rate, and ${\gamma }_{q}$ is the discount factor. The boosting process continues for up to 300 iterations or terminates early if no improvement in training MAPE is observed after 30 consecutive rounds.

### Stacking ensemble

Stacking is a heterogeneous ensemble learning technique that combines predictions from multiple base models using a meta-model trained on their outputs ^54^. Unlike homogeneous techniques like bagging and boosting, which aggregate outputs directly, stacking leverages diverse learning and models the relationships among the predictions through a secondary learner^55^. In a typical stacking framework, the base learners form the first layer (level 0), while the meta-learner constitutes the second layer (level 1). The base learners are independently trained on the dataset, and their predictions are used as inputs to train the meta-model^56^. For this study, the dataset $\mathcal{D}={\left\{\left({\mathbf{x}}_{i},{y}_{i}\right)\right\}}_{i=1}^{N}$ was randomly split into training and test subsets. The input feature matrix $\mathbf{X}\in {\mathbb{R}}^{n\times d}$ and target vector $\mathbf{y}\in {\mathbb{R}}^{n}$ were standardized prior to modelling. Four heterogeneous base learners were employed: Random Forest, Support Vector Regression (SVR), Extreme Gradient Boosting (XGBoost), and K-Nearest Neighbors (KNN).

The Random Forest model constructs $B$ decision tress ${\left\{{T}_{b}\right\}}_{b=1}^{B}$ on bootstrap samples and predicts the average output:

$${\widehat{y}}^{\text{RF}}(\mathbf{x})=\frac{1}{B}\sum_{b=1}^{B} {T}_{b}(\mathbf{x})$$

The Support Vector Regression (SVR) model minimizes a regularized loss function with an $\in$-insensitive margin, subject to constraints that penalize deviations exceeding $\in$= 0.1:

$$\underset{\mathbf{w},b,{\xi }_{i},{\xi }_{i}^{\xi }}{min} \frac{1}{2}\| \mathbf{w}{\| }^{2}+C\sum_{i=1}^{n} \left({\xi }_{i}+{\xi }_{i}^{*}\right)$$

For XGBoost, the model incrementally adds regression trees to minimize a regularized objective function: ${\mathcal{L}}^{(t)}=\sum_{i=1}^{n} {\ell}\left({y}_{i},{\widehat{y}}_{i}^{(t-1)}+{f}_{t}\left({\mathbf{x}}_{i}\right)\right)+\Omega \left({f}_{t}\right)$

Where the regularization term is $\Omega (f)=\gamma T+\frac{1}{2}\lambda \| w{\| }^{2}$. Model parameters included 200 trees, a learning rate of 0.1, and a maximum tree depth of 3.

The KNN model computes predictions as the average output of the *k* = 5 nearest neighbours:

$${\widehat{y}}^{\text{KNN}}(\mathbf{x})=\frac{1}{k}\sum_{{\mathbf{x}}_{j}\in {N}_{k}(\mathbf{x})} {y}_{j}$$

All base learners were trained in parallel on the training set. Their predictions were concatenated to form a new feature representation, which was further augmented with the original input features. This augmented dataset was then used to train the meta-learner. The meta-learner was an Elastic Net regressor, which combines ${{\ell}}_{1},{{\ell}}_{2}$ regularization to achieve a balance between feature sparsity and coefficient shrinkage.

### SHapley additive exPlanations (SHAP)

SHAP (SHapley Additive exPlanations) is a method rooted in cooperative game theory principles and is used to quantify the contribution of each input feature to a model’s prediction^57^. It adopts an additive feature attribution approach, where the model output is represented as a linear combination of the effects of each input variable^58^. In this study, SHAP values were employed to interpret the GrowNet model and to evaluate the influence of the individual soil index properties on the predicted residual friction angle.

For a predictive model $\widehat{y}(\mathbf{x})$, SHAP decomposes the prediction into:

$$\widehat{y}(\mathbf{x})={\phi }_{0}+\sum_{j=1}^{M} {\phi }_{j}$$

where ${\phi }_{0}={\mathbb{E}}_{\mathbf{x}}[\widehat{y}(\mathbf{x})]$ is the expected prediction over the training data, ${\phi }_{j}$ is the contribution of the $j$-th feature ${{\varvec{x}}}_{j}$, $M$ is the total number of features. 

Since GrowNet consists of an ensemble base learner (XGBoost) and a residual learning network, the total SHAP value for a feature ${{\varvec{x}}}_{j}$ is computed as the sum of its contributions to both components:

$${\phi }_{j}^{\text{GrowNet }}={\phi }_{j}^{\text{XGB}}+{\phi }_{j}^{\text{NN}}$$

The SHAP values for the XGBoost component of the GrowNet model, denoted as ${\phi }_{j}^{\text{XGB}}$, were computed using TreeExplainer, which efficiently leverages the decision tree structure. Conversely, the values for the residual neural network component, denoted as ${\phi }_{j}^{\text{NN}}$, were approximated using KernelExplainer, a model-agnostic method based on sampling. To quantify the overall influence of each soil index property on the prediction of the residual friction angle, the mean absolute SHAP value across the dataset was calculated as:

$$\text{Importance}\left({x}_{j}\right)=\frac{1}{n}\sum_{i=1}^{n} \left|{\phi }_{j}^{(i)}\right|$$

where ${\phi }_{j}^{(i)}$ is the SHAP value of feature ${{\varvec{x}}}_{j}$ for the *i-th* instance. This metric captures global feature importance by averaging the magnitude of each feature’s contribution, regardless of the direction (positive or negative), thereby identifying the most influential soil index properties in the model.

### Local interpretable model-agnostic explanations (LIME)

To complement the global feature attributions provided by SHAP, Local Interpretable Model-agnostic Explanations (LIME) was employed to investigate local interpretability of the GrowNet model. LIME approximates the complex behaviour of a black-box model ***f*** around a specific instance ***x*** by fitting a simple interpretable surrogate model. Given a prediction $f(\mathbf{x})$, LIME perturbs the input ${\varvec{x}}$ to generate a neighborhood ${\mathcal{Z}}_{x}$ and assigns a proximity weight ${\pi }_{{\varvec{x}}}(z)$ to each sample point $z\in {\mathcal{Z}}_{\mathbf{x}}$. It then solves the following optimization problem:

$$\text{arg}\underset{g\in G}{min} \mathcal{L}\left(f,g,{\pi }_{\text{x}}\right)+\Omega \left(g\right)$$

where where $g\in G$ is an interpretable model, $\mathcal{L}\left(f,g,{\pi }_{\text{x}}\right)$ measures the fidelity of $g$ to the original model $f$ in the local neighborhood, ${\pi }_{\text{x}}$ is an exponential kernel centered at $x$**,** $\Omega (g)$ is a regularization term that penalizes model complexity. The resulting local explanation is a sparse linear model of the form:

$$f(\mathbf{x})\approx g(\mathbf{x})={\beta }_{0}+\sum_{j\in S} {\beta }_{j}{x}_{j}$$

where $S\subset \left\{1,\dots ,M\right\}$ is a subset of the most influential features for that particular instance. By applying LIME, the study enabled case-specific interpretation, identifying the soil properties that had the most impact on the predicted residual friction angle for each observation.

### Model evaluations

The predictive performance of all three models was assessed using three standard error metrics: Root Mean Squared Error (RMSE), Mean Absolute Error (MAE), and the Coefficient of Determination (R^2^). These metrics were calculated separately for the training and test datasets to evaluate both model fit and generalization capacity.

The Root Mean Squared Error (RMSE), is a commonly used scale-dependent metric that provides a direct interpretation of the prediction error in the same units as the target variable. It is computed as:

$$\text{RMSE}=\sqrt{\frac{1}{n}\sum_{i=1}^{n} {\left({y}_{i}-{\widehat{y}}_{i}\right)}^{2}}$$

where ${y}_{i}$ and ${\widehat{y}}_{i}$ are the observed and predicted values, respectively, and $n$ is the number of samples. 

The Mean Absolute Error (MAE) quantifies the average magnitude of prediction errors without squaring the residuals, making it less sensitive to outliers than the Root Mean Square Error (RMSE). MAE is defined as:

$$\text{MAE}=\frac{1}{n}\sum_{i=1}^{n} \left|{y}_{i}-{\widehat{y}}_{i}\right|$$

To evaluate the proportion of variance explained by the models, the Coefficient of Determination (R²) was calculated:

$${R}^{2}=1-\frac{\sum_{i=1}^{n} {\left({y}_{i}-{\widehat{y}}_{i}\right)}^{2}}{\sum_{i=1}^{n} {\left({y}_{i}-\overline{y }\right)}^{2}},$$

where $\overline{y}$ is the mean of the observed values. An ${R}^{2}$ value close to 1 indicates stronger predictive performance and high explanatory power of the model.

Together, these metrics provide a comprehensive framework for evaluating model accuracy, robustness, and generalizability, thereby supporting the comparative analysis and validation of all three model configurations.

## Results and discussions

### Model performance evaluation

Table 3 summarizes the predictive performance of the GrowNet, RL-GBM, and Stacking models in estimating the residual friction angle, based on 320 random sampling iterations. Among the models assessed, the GrowNet hybrid model demonstrated superior generalization capability and predictive accuracy. It produced R^2^ values of 0.97 (training) and 0.94 (testing), coupled with the lowest RMSE (1.16 training, 1.87 testing) and MAE (90.82 training, 1.17 testing).

**Table 3:** Performance Evaluation of Machine Learning and Empirical Models for Predicting Residual Friction Angle (Φ_r_).

Model/Equation	*R*^*2*^		RMSE		MAE	
	Training	Testing	Training	Testing	Training	Testing
GrowNet	0.97	0.94	1.16	1.87	0.82	1.17
RL-GBM	0.96	0.92	1.44	2.10	1.08	1.46
Stacking	0.89	0.84	2.36	3.04	1.74	2.14
Skempton^33^ φr = 31.4—11.6log(CF)	-	0.52	-	5.27	-	3.99
Voight^32^ φr = 46.6—23.2log(PI)	-	0.43	-	5.79	-	4.55
Wesley^29^ φr = 30—14.33log(PI)	-	0.07	-	7.89	-	6.14

The RL-GBM hybrid model also performed well with R^2^ values of 0.96 (training) and 0.92 (testing). However, its error metrics (RMSE: 1.44 training, 2.10 testing; MAE: 1.08 training, 1.46 testing) were slightly higher than GrowNet, suggesting slightly reduced predictive accuracy and generalization capability.

In contrast, the Stacking ensemble model showed comparatively weaker performance. Specifically, R^2^ decreased from 0.89 (training) to 0.84 (testing), and error metrics increased (RMSE: 2.36 to 3.04; MAE: 1.74 to 2.14). This drop in performance indicates a reduced ability to capture the underlying relationships in the dataset.

**Fig. 4:**
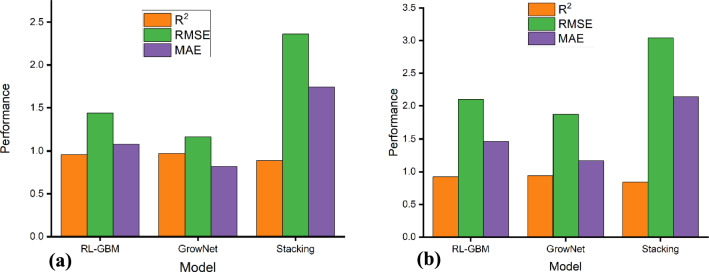
Comparative Performance of the Machine Learning Models for Predicting Residual Friction Angle (φ_r_): (**a**) Training set; (**b**) Testing set.

**Fig. 5:**
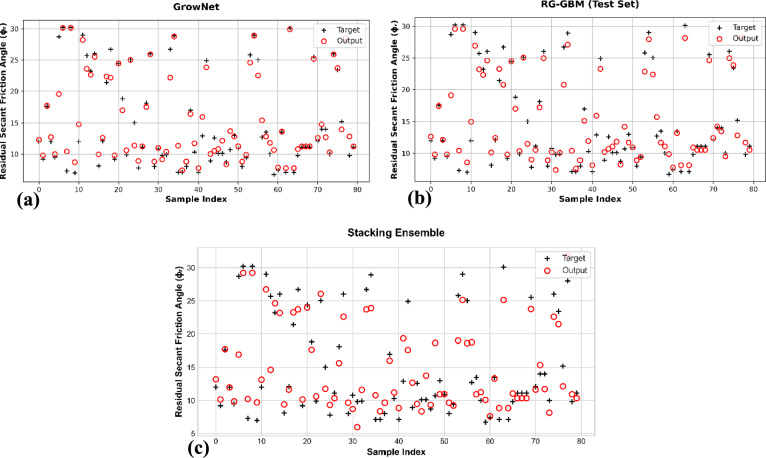
Predicted Versus Experimental Residual Friction Angle (Φ_r_) For The Machine Learning Models: (**a**) GrowNet; (**b**) RG-GBM; and (**c**) Stacking Ensemble.

As illustrated in Fig. 4, the GrowNet model consistently outperforms the alternatives across all performance metrics in both training and testing. The stability of the GrowNet model is also supported by Fig. 5, which shows a narrow spread of prediction errors across the test cases, with the RL-GBM also outperforming the stacking ensemble. These results indicate a strong agreement between experimental measurements and model predictions, highlighting GrowNet’s ability to capture complex, nonlinear relationships in geotechnical datasets. Moreover, the model’s performance showed minimal variability across 320 random sampling iterations, further reinforcing its robustness and reliability in predicting the residual friction angle of clay soils.

#### Statistical significance testing

The Wilcoxon signed-rank test was applied to the residuals obtained from the test dataset to evaluate the statistical significance of the observed differences in predictive performance among the machine learning models. This non-parametric test is particularly well suited for paired comparisons and is robust against deviations from normality in the error distributions, an issue commonly encountered in geotechnical datasets. As shown in Table 4, the results highlight three key comparisons. First, GrowNet demonstrated statistically significant superiority over RLGBM (W = 805, p < 0.001 for both RMSE and MAE). Second, the GrowNet performance advantage was even more pronounced when compared to the Stacking ensemble (W = 465, p < 0.001). Third, RLGBM also significantly outperformed the Stacking approach (W = 658, p < 0.001). These consistently low p-values (all p-values < 0.001) confirm that the observed differences in predictive accuracy are statistically significant and underscore the genuine superiority of the GrowNet model in estimating residual friction angle in clay soils.

**Table 4:** Wilcoxon Signed-Rank Test Results Comparing Model Prediction Errors (RMSE And MAE).

Model Comparison	Error Metric	GrowNet	RLGBM	Stacking	Test Statistic (W)	p-value	Significant (p < 0.05)
GrowNet vs. RLGBM	RMSE	1.87	2.10	-	805	0.0001	Yes
	MAE	1.17	1.46	-	805	0.0001	Yes
GrowNet vs. Stacking	RMSE	1.87	-	3.04	462	0.0000	Yes
	MAE	1.17	-	2.14	462	0.0000	Yes
RLGBM vs. Stacking	RMSE	-	2.10	3.04	658	0.0000	Yes
	MAE	-	1.46	2.14	658	0.0000	Yes

#### Practical applicability of the developed models

To assess the practical applicability of the developed machine learning models, their predictive performance was compared with widely used correlations for estimating residual friction angle, specifically those proposed by Skempton^33^, Voight^32^, and Wesley^29^ equations. The results of this comparison, presented in Table 3, demonstrate that all the empirical models have significantly lower predictive accuracy when applied to the testing dataset. The Skempton^33^ equation produced an R^2^ of 0.52, RMSE of 5.27, and MAE of 3.99. The Voight^32^ equation produced a slightly worse performance compared to the Skempton^33^ equation, with an R^2^ of 0.43, RMSE of 5.79, and MAE of 4.55. In contrast, the Wesley^29^ equation performed very poorly on the dataset, producing an R^2^ of 0.07, RMSE of 7.89, and MAE of 6.14. The foregoing results (Table 3) show that although these empirical correlations are commonly adopted due to their simplicity and ease of use, they are inherently limited in capturing complex, nonlinear interactions among multiple geotechnical variables.

Based on the comparison provided in Table 3, all the machine learning models developed in this study, including the RLGBM and the stacking ensemble, outperformed the considered empirical models. These findings underscore the need for adopting data-driven approaches in modeling residual shear strength. Consequently, the developed machine learning models, especially GrowNet, represent a practical advancement in residual friction angle prediction accuracy.

### Sensitivity analysis

This section presents a comprehensive sensitivity analysis to evaluate the influence of soil index properties on the residual shear strength of clay soils, using the high-performing GrowNet model as the analytical framework. Two advanced explainable artificial intelligence (XAI) techniques, SHAP (SHapley Additive exPlanations) and LIME (Local Interpretable Model-agnostic Explanations), are employed to provide both global and local interpretations of feature importance.

**Fig. 6:**
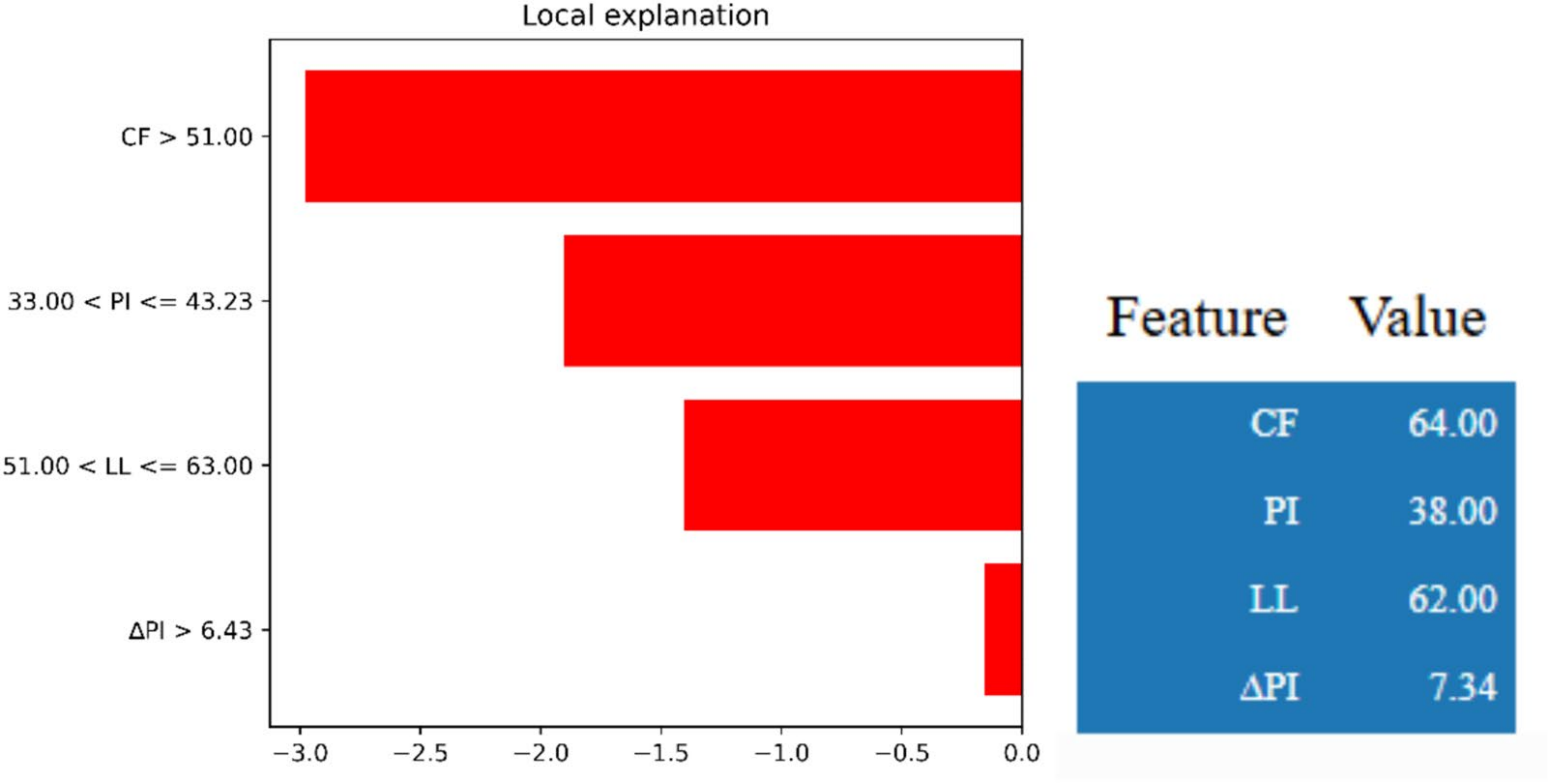
Absolute contribution of each input feature to model prediction using LIME.

The local interpretability results, obtained through LIME and illustrated in Fig. 6, provide insights into the impact of individual features for a representative prediction. The associated input values are clay fraction (CF) of 64.00%, plasticity index (PI) of 38.00%, liquid limit (LL) of 62.00%, and ΔPI of 7.34. Among these, CF > 51.00% exerts the most significant negative influence on the predicted residual friction angle (φ_r_), followed by PI in the range 33.00–43.23, LL between 51.00–63.00, and ΔPI > 6.43. This localized explanation shows that, for this specific prediction, high clay content is the most critical factor contributing to the reduction of the residual friction angle (φ_r_).

**Fig. 7:**
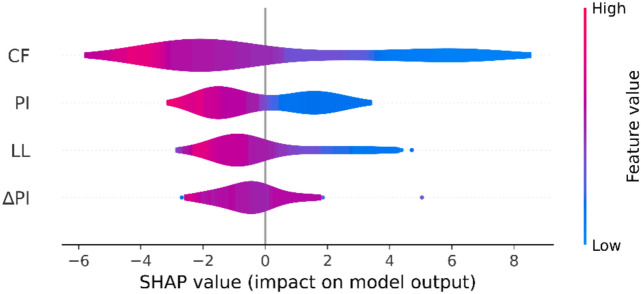
Global SHAP Summary Plot Showing the Relative Importance of Input Variables in Predicting Residual Friction Angle.

**Fig. 8:**
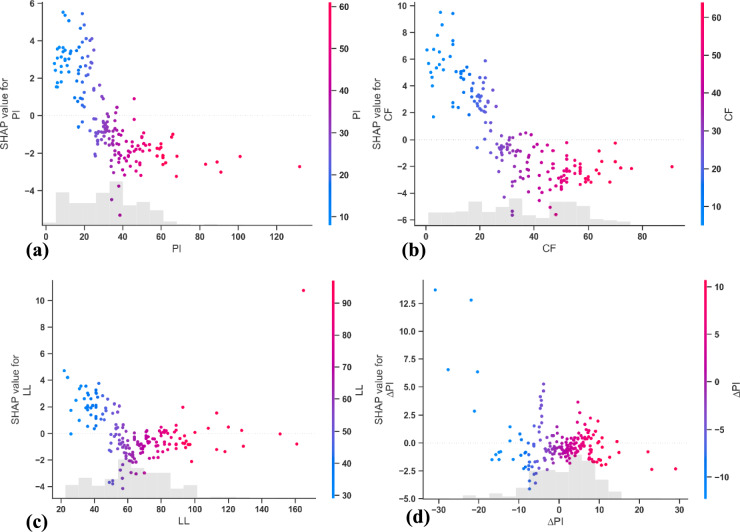
SHAP Dependence Plots for Input Variables: (**a**) Plasticity index (PI); (**b**) Clay fraction (CF); (**c**)Liquid limit (LL); and (**d**) Change in plasticity index (ΔPI).

Complementary global analysis using SHAP, shown in Fig. 7, confirms the overall importance of the clay fraction across the entire dataset. The SHAP summary plot ranks clay fraction as the most influential feature, followed by plasticity index, liquid limit, and change in plasticity index (ΔPI). As depicted in Fig. 8, the probability of predicting a high value of RSS decreases significantly as the values of the CF and PI increase. Notably, the SHAP value distribution indicates that the highest values of these distributions are consistently associated with a decrease in the residual friction angle, as evidenced by the clustering of high feature values (in red) towards negative SHAP values. This inverse relationship is observed across all four input variables and suggests a consistent trend where increases in these index properties correspond to reductions in residual shear strength. The alignment between LIME and SHAP results reinforces the reliability and interpretability of the GrowNet model, both locally and globally. These findings are in agreement with prior studies, which have also identified clay fraction as the principal factor influencing the residual strength behaviour of fine-grained soils ^25,37^. Overall, the XAI-enhanced sensitivity analysis offers a transparent and mechanistic understanding of the soil behaviour in residual conditions, confirming that clay-rich high-plasticity soils exhibit lower residual friction angles due to their microstructural and mineralogical properties.

## Conclusion

This study investigated the prediction and interpretability of residual shear strength, a critical parameter in geotechnical analysis, particularly for landslide reactivation. Three advanced machine learning approaches, GrowNet, Reinforcement Learning Gradient Boosting Machine (RL-GBM), and Stacking Ensemble, were developed and evaluated to enhance prediction accuracy. To ensure model transparency and practical applicability, Explainable Artificial Intelligence (XAI) techniques, SHAP (SHapley Additive exPlanations) and LIME (Local Interpretable Model-agnostic Explanations) were employed to interpret the predictions.

A harmonized dataset comprising 400 samples was used, incorporating four easily obtainable soil index properties (Liquid Limit, Clay Fraction, Plasticity Index, and Change in Plasticity Index) as input features, with residual friction angle as the target variable. Among the models developed, GrowNet demonstrated superior performance with the highest predictive accuracy and lowest error metrics, outperforming both the RL-GBM and the Stacking Ensemble models. The RL-GBM algorithm showed good generalization performance, while the Stacking Ensemble yielded relatively weaker results.

The GrowNet model was selected for XAI analysis due to its exceptional predictive accuracy. Interpretability results from SHAP and LIME analyses consistently identified Clay Fraction as the most influential parameter affecting the residual friction angle, followed by Plasticity Index. SHAP dependency plots further revealed non-linear relationships and threshold effects, such as a marked increase in influence of clay fraction when exceeding 50%, which has direct implications for evaluating soil behavior in high-clay-content slopes. The study demonstrates that hybrid machine learning techniques like GrowNet, when paired with robust interpretability frameworks, can substantially enhance the prediction of residual shear strength in clay soils. These models provide a cost-effective, time-efficient, and accurate alternative to traditional laboratory testing, with direct applications in landslide hazard assessment, slope stability analysis, and geotechnical design in landslide-prone areas.

Nonetheless, this study has limitations, including reliance on a fixed dataset, exclusion of mineralogical or stress history data, and limited external validation. Future research should focus on expanding datasets, integrating mineralogical and site-specific information, and applying advanced modeling techniques to further improve prediction accuracy and generalizability across diverse geotechnical conditions.

## Statements and Declarations

## Data availability statement

The datasets generated during and/or analysed during this work are available from the corresponding author upon reasonable request.
